# Multivariable Heuristic Approach to Intrusion Detection in Network Environments

**DOI:** 10.3390/e23060776

**Published:** 2021-06-19

**Authors:** Marcin Niemiec, Rafał Kościej, Bartłomiej Gdowski

**Affiliations:** AGH University of Science and Technology, Department of Telecommunications, Mickiewicza 30, 30-059 Krakow, Poland; kosciej.rafal@gmail.com (R.K.); b.gdowski620@gmail.com (B.G.)

**Keywords:** cybersecurity, intrusion detection, network attack, heuristic algorithm, flags, entropy

## Abstract

The Internet is an inseparable part of our contemporary lives. This means that protection against threats and attacks is crucial for major companies and for individual users. There is a demand for the ongoing development of methods for ensuring security in cyberspace. A crucial cybersecurity solution is intrusion detection systems, which detect attacks in network environments and responds appropriately. This article presents a new multivariable heuristic intrusion detection algorithm based on different types of flags and values of entropy. The data is shared by organisations to help increase the effectiveness of intrusion detection. The authors also propose default values for parameters of a heuristic algorithm and values regarding detection thresholds. This solution has been implemented in a well-known, open-source system and verified with a series of tests. Additionally, the authors investigated how updating the variables affects the intrusion detection process. The results confirmed the effectiveness of the proposed approach and heuristic algorithm.

## 1. Introduction

The ongoing evolution of science and technology continues to bring new challenges. With the Internet becoming one of the most important inventions of the last century and an integral part of today’s world, new threats are emerging [1]. People are increasingly using the Internet for tasks that would have traditionally been done in person. Convenient online payments have convinced many to go online, even for the simplest activities. Remote working is now a common element of corporate network infrastructure. However, conducting our everyday lives online means that less-careful users are at risk of cyberattack [2]. Harmful software, viruses, and many other means of hacking are constantly being developed [3,4]. Vulnerabilities could mean losses for individual users, but they could also extend to millions or even billions of dollars [5,6]. This drives the development of effective security tools. Companies and individual users use a range of security tools to protect themselves against network attacks [7]. These tools should detect unexpected activities in the network and allow users to take appropriate action. The growing number of new and unknown attacks means that new methods of attack detection are required.

Awareness of the importance of cybersecurity encourages organisations to engage in joint defence activities, particularly those operating in the same sector, such as energy, healthcare, etc. By working together, they are able to collect and process data regarding sector-specific attacks and malicious software [8,9]. Multisector and multidomain collaboration is also frequently required. This is consistent with the development of cybersecurity in the EU, where the Horizon 2020 projects brings together partners to establish the European Cybersecurity Competence Network [10]. The ECHO project [11] is a good example of such a broad collaboration.

The federated approach to cybersecurity helps detect new network attacks and protect companies’ assets more efficiently. Data collected and processed by federated entities can be used by all members to improve the effectiveness of intrusion detection. In this paper, the authors introduce a new multivariable heuristic intrusion detection algorithm based on shared data. Such a joint approach to attack and malware detection allows the federated companies to share knowledge and make the best decisions regarding suspicious traffic.

The remainder of the paper proceeds as follows: Related work is reviewed in Section 2. Section 3 provides an introduction to detecting intrusions in cyberspace. A novel multivariable heuristic intrusion detection algorithm is presented in Section 4. The section also proposes a format of shared data and types of flags. The experimental results are presented and discussed in Section 5. Finally, Section 6 concludes the paper.

## 2. Related Work

Two main types of intrusion detection algorithms can be distinguished: an approach based on the predefined attack’s signature [12] and methods analysing behaviours to detect anomalies [13,14]. The second group contains heuristic algorithms reviewed by Kenny et al. in [15]. The list includes most types of algorithms proposed in the research on heuristic intrusion detection. Ali and Malebary [16] introduced a solution based on particle swarm optimisation (PSO)—an intelligent phishing website detection in the form of feature weighting. For five out of six common machine learning algorithms, the proposed method achieved better detection accuracy than other feature selection and weighting methods mentioned in the paper. Jacob [17] proposed a tabu search algorithm—automatic signature generation for detected cross-site scripting (XSS) attacks. Although the true positive ratio of the solution is acceptable, the detection algorithm is focused on finding XSS specific keywords instead of looking for injection patterns. Yerong et al. [18] combined two heuristic methods in their research—the support vector machine (SVM) used for intrusion detection was optimised using a genetic algorithm. The optimisation indeed improved detection accuracy and decreased the number of false positives compared to the values obtained by a radial basis function neural network and unoptimised SVM. Jothi et al. [19] provided yet another type of heuristic solution—an artificial neural network (ANN). The authors implemented an accurate machine learning model for the detection of structured query language (SQL) injection attacks, which could be implemented, for example, to prevent attacks during login sessions.

Most of the papers on heuristic intrusion detection have focused on machine learning [20,21,22,23,24,25,26,27,28,29]. The authors of this paper consider a different approach to intrusion detection: packet scoring. The topic has been studied by Subburathinam and Saravanan [30], who proposed calculating the score of the packet depending on different variables, e.g., port number or protocol. At the same time, the conditional legitimate probability was being checked—if either score or probability was an anomaly, the packet was dropped. Murtuza and Asawa [31] introduced the fitness score used for distributed denial of service (DDoS) detection in software-defined networks (SDN). The fitness value for each packet was either incremented or decremented depending on, for example, previous successful connections or protocol. Then, the packet was categorised depending on its score and processed further. Prasath and Perumal [32] also presented a heuristic algorithm for intrusion detection in SDN networks. However, this method is focused on finding anomalies using extracted features of flows, e.g., duration, protocol type, service. It is worth mentioning that the large number of features can decrease the performance of intrusion detection significantly [33,34]. In [35], Mukhopadhyay et al. proposed a lightweight heuristic intrusion detection and prevention system; its decision making engine is based on frame data and source/destination addresses. The decision engine can also take into account selected external data, such as the reputation of a given URL. The solution presented in this paper extends such an approach into different flags regarding suspicious/malicious IP addresses. The idea to include fuzzy entropy as a feature to support intrusion detection based on machine learning methods was introduced by Varma et al. [36]. The feature extraction method based on the regularised correntropy criterion was also proposed by Xing and Ren [37]. However, this paper assumes the direct impact of entropy to the score of a given packet.

Aside from packet scoring, the authors of this paper also focus on a federated approach to intrusion detection. The proposed solution operates on a shared file with malicious addresses, assigned flags, and entropy values, which could be updated by federated entities in the case of new attacks and sent out to all members of the federation to ensure security.

## 3. Intrusion Detection

The evolution of malware and emerging new attacks are driving the development of attack detection methods [38]. These methods should provide effective protection to users/companies and their data against intruders [39]. The detection methods can focus on analysing the behaviour of network traffic and detecting anomalies that are known or that could be a new type of attack on network infrastructure. The assumptions of such a solution are highly restrictive, since it should return low numbers of false positives and false negatives. If such defects are high, the system administrator may become complacent and fail to respond to a real attack [40]. Suitable detection methods should also be efficient enough to process network traffic and inform the administrator of any potential threats as soon as possible.

### 3.1. Intrusion Detection Systems

An intrusion detection system (IDS) is a solution that is used to monitor network traffic and able to detect attacks [41]. This solution is mainly used in two ways: as a network-based IDS located behind a firewall to analyse incoming traffic, or as a host-based IDS to analyse traffic targeted to specific host.

It is worth mentioning that IDS conducts a significantly more advanced analysis than a typical firewall, which is a filtering point between a local and external network. The major task of a firewall is to allow or deny network traffic using static analysis. Therefore, a firewall’s configuration rules focus mainly on source/destination IP addresses or ports, which may prevent the firewall from detecting malicious traffic [42]. Firewalls frequently do not conduct analysis as advanced as IDS; however, a combination of these two solutions provides more efficient protection to network infrastructure [43].

The placement of an IDS is critical and varies depending on what the user needs to protect. It is crucial that the balance between network performance and the range of IDS operation is maintained. The most obvious placement of an IDS is behind the firewall, allowing for monitoring of the entire network; however, this could create a bottleneck that may decrease the overall throughput of the network. On the other hand, if the IDS is placed deeper inside the network, the performance levels will be maintained, while a part of the network will be left vulnerable [44,45].

There are two main types of IDS: software solutions (e.g., Snort [46] or Suricata [47]) and hardware solutions (e.g., devices developed by Cisco Systems [48] or Palo Alto Networks [49]). Selecting the most appropriate solution depends on infrastructure, budget, and other specific requirements of cybersecurity staff. Optimal IDS deployment and configuration make it possible for the network to stay hidden from attackers while remaining transparent to network users. However, it is a key element of network security, providing a response to any attack.

### 3.2. Detection Methods

The key purpose of IDS is to detect unwanted traffic in the network that could be a potential attack [50]. There are two main types of detection techniques [51]: misuse detection and anomaly detection.

Misuse detection is based on the attack’s signature. This type of detection uses a predefined attack signature and compares it with an analysed packet or groups of packets [52]. If the signature or part of it matches the malicious signature, the event is reported. Misuse detection is effective and produces low levels of false negatives and false positives. However, this solution cannot detect new types of attack, which are unlikely to match any known signatures. Therefore, if the attack differs from the signature even slightly, it is not detected. This makes it essential for the producer/vendor of the IDS to update the signatures database frequently.

Anomaly detection, also known as behaviour-based detection, assumes that behaviour that determines the attacker’s likely activity is different from the behaviour of a permitted network user [53]. IDS supporting anomaly-based detection is highly effective at finding zero-day attacks; however, it generates high volumes of false positives. We can distinguish two types of behaviour-based attack identification: heuristic analysis and anomaly analysis. The first type is based on potential behaviours, which can occur with different kind of attacks, such as port scanning or unauthorised access to confidential resources. The second type relies on anomaly recognition by detecting unusual activities. For instance, if a user logs into the local database outside of their usual hours and tries to access confidential data, it may be seen as an anomaly. The heuristic approach can also be based on data shared by the federated organisations.

## 4. Multivariable Heuristic Approach

A joint approach to attack detection is more effective than an individual approach, encouraging companies working in a given sector to create federations. This approach means each member of the federation has access to a broad knowledge of threats in cyberspace. However, shared data regarding malicious or suspicious entities can be fragmented and covers a range of aspects of the threat. Such inconsistent data should be organised into groups.

### 4.1. Flags

Groups known as flags describe the nature of a given threat. This information can be shared across the federation and is used by a heuristic detection algorithm. The choice of flags was inspired by the common vulnerability scoring system (CVSS) and authors’ practical knowledge—including joint work in the H2020 ECHO project [11]. Parameters and their values can be configured depending on the local security policy. Flags and their default values are described below. These values were chosen for the purpose of the test to present functionality of the algorithm.

- **dangerous**—This flag identifies the severity of the threat associated with an IP address (Table 1). The value of this flag is subjective and depends on the environment/federation. In some cases, the attack may not be especially harmful. For example, a phishing attack on medical wristband infrastructure is not especially dangerous; on the other hand, the same type of attack on corporate infrastructure can be critical. The value of this flag and the decision of which flag to assign to the given IP address can be based on an analysis of other flags.
- **attack**—This flag specifies the type of attack in which the IP address was recently involved. The value of this flag may differ from its environment because the effectiveness of an attack also depends on the network’s purpose and users. Table 2 shows descriptions and default values for attack flags.
- **range**—This flag describes the impact of an attack by an IP address on other network components such as the server, switch, or router. In this case, a given attack may affect only a single attacked network component or spread over a part or all of the infrastructure. Table 3 shows description and default values for range flags.
- **access**—Some attacks (e.g., phishing, malware) require user action within the network, while others (e.g., DDoS, DoS) do not require user response. This type of flag describes the need for user response within the network. Table 4 shows two possible flags: none and user. The first describes a situation when the attack does not require a user response. The second flag describes a situation when the attack requires a user response (e.g., opening an attachment in an email).
- **availability**—Some attacks, such as ransomware, cause a partial or complete loss of access to the unit and data on it. This type of flag describes the impact on the availability of the attacked component. Table 5 shows three levels of impact on the functionality of a given component in the network.

### 4.2. Entropy

Entropy is a concept derived from information theory. Entropy, introduced by Claude Shannon, is the average amount of information carried by a single message [54]. By defining the probability of an event, it can be determined whether the event is recurring or rare. With regard to a computer network, the entropy of a phenomenon can determine whether it is a desired activity in a given network or an anomaly [55,56].

Assume that X is a discrete random variable, with a probability distribution $p\left(x_{1}\right),p\left(x_{2}\right),\ldots ,p\left(x_{i}\right),$. Each probability $p(x_{i})$ meets the condition (1).

(1) $$0\leq p\left(x_{i}\right)\leq 1,\sum_{i=1}^{n}p\left(x_{i}\right)=1$$

For the assumed condition, the entropy takes the Formula (2).

(2) $$H_{s}\left(X\right)=\sum_{i=1}^{n}p\left(x_{i}\right)log\frac{1}{p(x_{i})}=-\sum_{i=1}^{n}p\left(x_{i}\right)logp(x_{i})$$

### 4.3. Shared Data

The format of data shared in the federation should be simple and scalable. As we are operating on a relatively small number of addresses, comma-separated values (CSV) format was used. This type of file is concise, readable, and can be formatted and edited easily with many applications, such as LibreOffice Calc, Microsoft Excel, and Notepad. While the CSV format is convenient for operation on small amounts of data, the file type could be changed to more compact and scalable format, e.g., JavaScript Object Notation (JSON) or Extensible Markup Language (XML).

Each record must contain the IP address of the suspicious/malicious entity, defined flags, and entropy value. These sections in one record can be separated by commas. The general structure of a single record is as follows:

IP_address,dangerous,attack,range,access,availability,counter,entropy

An example list with records in the correct format is presented below. This kind of list (CSV file) can contain thousands of records with suspicious/malicious addresses.

52.114.75.115,H,M,P,U,N,0,0.0

54.239.192.118,M,R,C,N,P,1200,4.777507

52.113.199.181,M,R,S,U,C,7679,2.103476

192.168.0.192,L,R,P,N,N,0,0.0

35.244.181.201,M,M,C,U,P,461,6.082576

The first section defines a malicious IP address. Such an address can be provided by another company that had been attacked and, following forensic analysis, is confirmed that it took part in the attack. The second section determines the severity of the threat associated with a given IP address (e.g., the flag set to High may mean the website where the ransomware was downloaded, while the flag set to Low may mean that the IP address that was involved in a DDoS attack is a bot). The third section describes the types of attacks in which this IP address was involved. The fourth section determines the range of the attack on local network infrastructure. The range flag determines how many stations could be attacked. The fifth section contains access flags, which mark the requirement for user action within the network. The sixth flag describes the impact on the availability of the attacked network component. Finally, the structure includes a counter of the address appearing in the network shown alongside the entropy value of this address in the local network. The default values of counter and entropy are equal to zero.

### 4.4. Detection Algorithm

The proposed multivariable heuristic algorithm should take into account the flags and entropy value. However, the entropy depends on the number of received packets from a given IP address; therefore, the final value should be calculated for each captured packet. Additionally, this value should depend on the value of each flag in the correct proportion. Thus, the following formula for calculating the packet value is used:

(3) $$\begin{array}{c} PV_{f}=PV_{i}+\left(\alpha *dangerous\right)+\left(\beta *attack\right)+\left(\gamma *range\right)+\left(\delta *access\right)+\\ (\epsilon *availability)+(\eta *entropy), \end{array}$$

where $PV_{f}$ is the final packet value and $PV_{i}$ is the initial packet value.

Parameters α, β, γ, δ, ϵ, and η should be chosen regarding the security policy. The authors suggest that the influence of the entropy value should be limited. Therefore, the η value has been set to 0.5 for calculations during the verification tests. Further, for dangerous and attack flags, it is a subjective assessment of how to evaluate the attack. Therefore, the ratio of 65% of the dangerous flag value and 35% of the attack flag value was adopted for calculations. The default parameters are shown in Table 6.

The final elements of the detection algorithm are related to the selected detection threshold. The heuristic algorithm should generate an alert if this threshold is exceeded. Therefore, three different detection parameters should be defined.

- **packet_value**—Initial value of the received packet immediately after the packet is captured. This value is the same for each analysed packet.
- **sensitivity**—Lower limit of the packet value. When this limit is exceeded (following analysis), the packet is reported to the console.
- **entropy**—Upper limit of the packet entropy value above which the packet is reported to the console.

Each of these parameters should have default values related to the deployed security policy in the protected network. Table 7 presents the proposed default values, which were selected during the experiments described in the next section.

## 5. Verification

The proposed multivariable heuristic detection algorithm must be verified in real-life scenarios. Therefore, this solution was tested in a network environment to detect malicious traffic. Additionally, the authors verified how updates of variables affect the efficiency and accuracy of the detection algorithm.

### 5.1. Methodology and Test Environment

The verification was performed in a Snort environment—an open source IDS network capable of logging and analysing incoming traffic in real-time. Snort is a powerful tool used to detect and prevent intrusions in networks [57]. It has been in use for over 20 years and is one of the most popular open source IDS tools [58]. However, this tool is a signature-based detection system. This means that new heuristic functionalities had to be implemented to verify the proposed multivariable heuristic detection algorithm.

The first step of detecting anomalies in Snort is collecting (sniffing) network traffic and identifying the structure of each packet. This requires a packet capture and filtering engine for acquiring data such as [59] packet capture time, length of the packet, size of the captured packet, and a pointer to the contents of the packet. After capturing the packet, Snort begins decoding: the acquired packet enters the packet decoder depending on the link layer from which it is read. Next, preprocessors expand Snort’s functionality by making it possible to easily configure the packet processing modules [60]. The preprocessors are an element of Snort, which is key when it comes to developing a new functionality inside the environmental engine. The authors developed and deployed two new preprocessors: one allowing Snort to collect and update variables regarding malicious IP addresses, and the other to update flags and entropy values in a dynamic manner.

Detection rules are an important element of Snort. A single rule consists of a header and options. The header contains the rule’s action, protocol type (currently supporting TCP, UDP, ICMP, and IP), destination IP addresses and netmasks, direction operator (used to indicate the direction of the traffic the rule applies to), and source and destination port information. Options contain alert messages and information that determine whether the rule action should be taken depending on the inspected packet [46]. Snort’s detection ability was expanded during the verification process based on rules focused on SQL Injection (SQLi). This type of attack exploits application security vulnerabilities to inject SQL queries into a database.

As mentioned, the new heuristic preprocessors in Snort add new functionality to this environment. The configuration file should contain a path to a CSV file with malicious IP addresses. Each address should have flags and an entropy value assigned to it. Each flag has a value that will be added to the packet rating. The flag values must be negative, so their absolute value will be subtracted instead. The evaluation of packets starts at a predefined packet_value variable. Depending on the flags and entropy assigned to the address, the packet rating is updated (hence, the negative values assigned to the flags). At the end, packet_value is compared to the sensitivity variable, which is a deciding factor in displaying alerts.

### 5.2. Validation of the Algorithm

This section presents the functional verification of the multivariable heuristic algorithm. To show how the algorithm operates in different environments, the selection of flags for IP addresses was random. Listing 1 presents the shared file containing information on malicious IP addresses and flags.

**Listing 1.** The content of a shared file (before test).

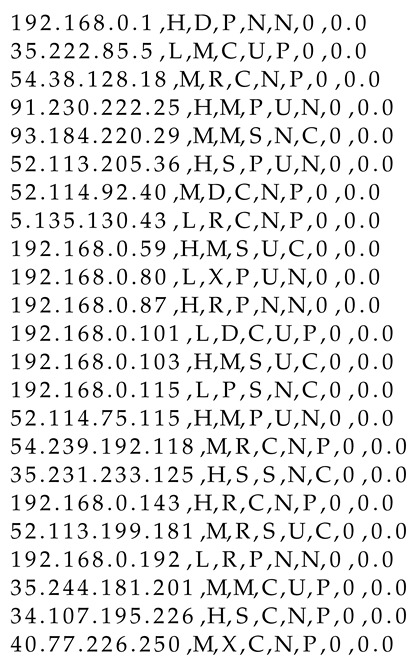



**Figure 1:**
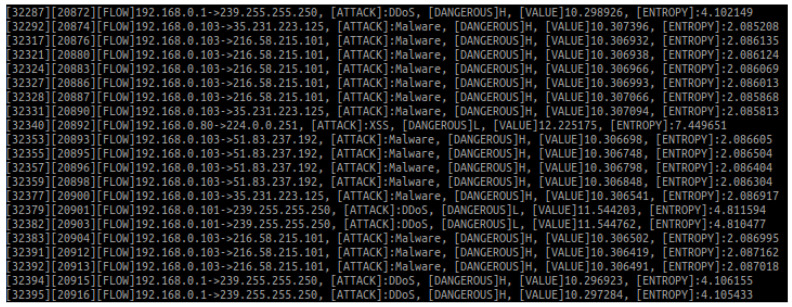
Logs received during the analysis.

During detection, logs related to individual packets can be seen in the console and are saved to a log file. The packets are processed to update the shared data for further usage by the federated entities. Figure 1 shows example logs that appear during the detection process. Each log contains selected flags (type of attack related to the given IP address and dangerous flag associated with the given IP address), package value after calculations based on Equation (3), and current entropy value for the specific malicious IP address.

**Figure 2:**
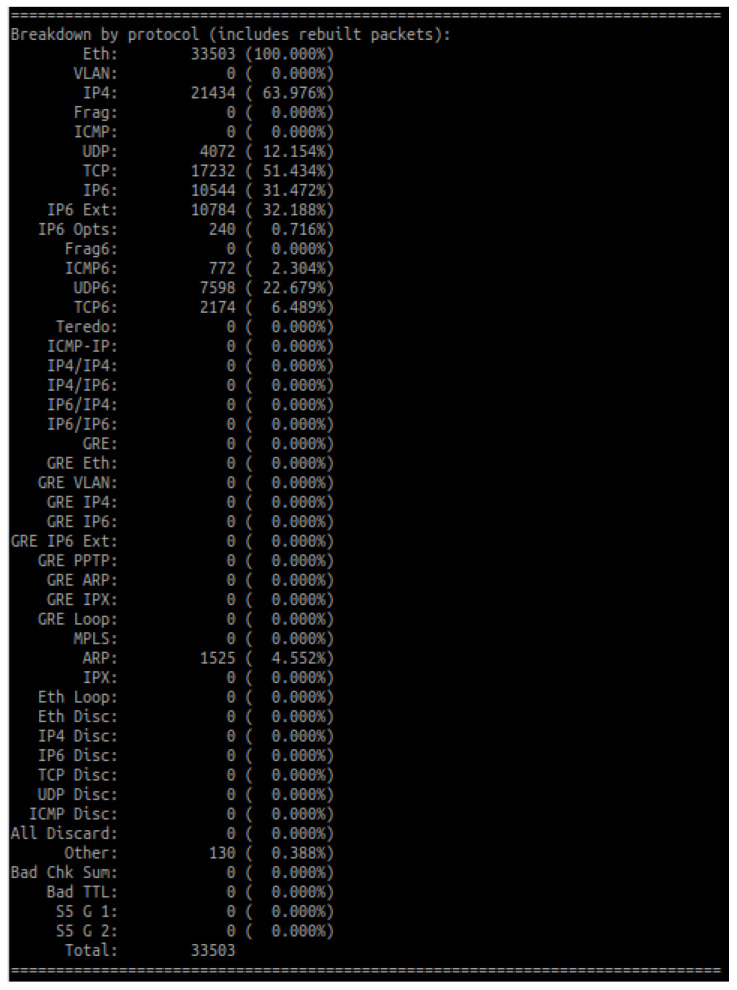
Number and percentage distribution of packets during the analysis.

During the verification test, 33,503 packets captured from a local network were analysed (there was no additional network traffic generated because of test’s purpose: functional verification of the proposed solution). The test was performed on a personal computer. Figure 2 shows a brief summary. Most of the traffic ran on IPv4. Listing 2 presents the shared file updated immediately after the test. The file contains updated data related to malicious IP addresses, showing significant changes compared with the status before the analysis.

**Listing 2.** The content of a shared file (after the test).

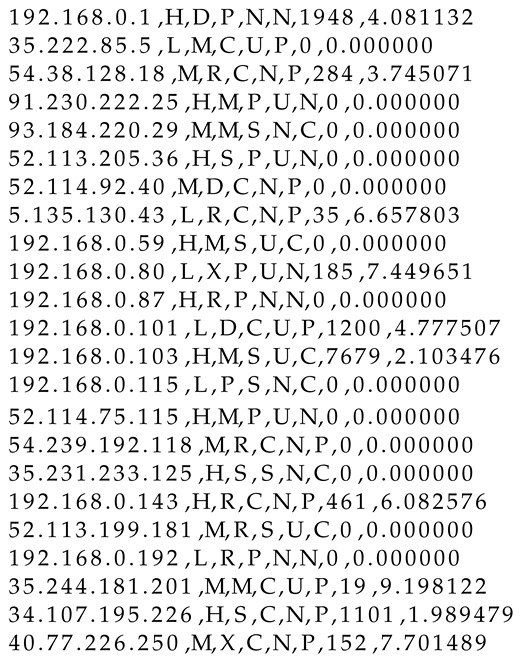



**Figure 3:**
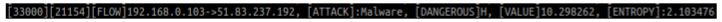
Entropy for the address 192.168.0.103.

In order to verify the algorithm operation, the packet value for the most frequent IP address (which is 192.168.0.103) has been calculated manually and then compared to the value computed by the algorithm. The calculations were based on Equation (3) and the default values of parameters (Table 6). Additionally, Figure 3 shows the log related to a packet from 192.168.0.103, which contains the packet value assigned by the detection algorithm.

192.168.0.103,H,M,S,U,C,7679,2.103476

(4) $$\begin{array}{c} PV_{F}=23+\left(0.65*\left(-6\right)\right)+\left(0.35*\left(-5\right)\right)-1-1-4-\left(0.5*\left(2.103476\right)\right)\\ =10.298262 \end{array}$$

The functional verification of the heuristic detection algorithm demonstrates that, based on the external shared data (flags and entropy), the packet value can be determined in quantitative way, as both values—calculated and computed—are equal. The decision is made when this value is compared with the threshold. This approach detects malicious traffic.

### 5.3. Updating of Variables

This test verifies how the duration of the detection process affects effectiveness. In this scenario, the authors used network traffic containing SQL Injection attacks (the environment SQLi-LABS [61,62] was used to validate these attacks) and wrote the *Iterate_Snort* script. As the name suggests, the script contains an iterational algorithm that works alternately with attack detection by Snort, based on its output, and prepares a file of malicious addresses. The main goals of this algorithm is to detect attacks (in this scenario, SQL Injections), collect information about specific IP addresses that have performed an attack, and update variables (e.g., flags) in the shared file.

The created script requires two arguments: iterate, which sets the number of iterations, and timer, which sets the duration of a single scanning iteration by Snort. Snort operates with the appropriate set of rules for SQLi detection and an option that allows the program to log the alerts into a specified folder. Then, another script is run to create updated CSV files based on the collected alerts. Both processes are repeated until the number of completed iterations is equal to iterate.

**Figure 4:**
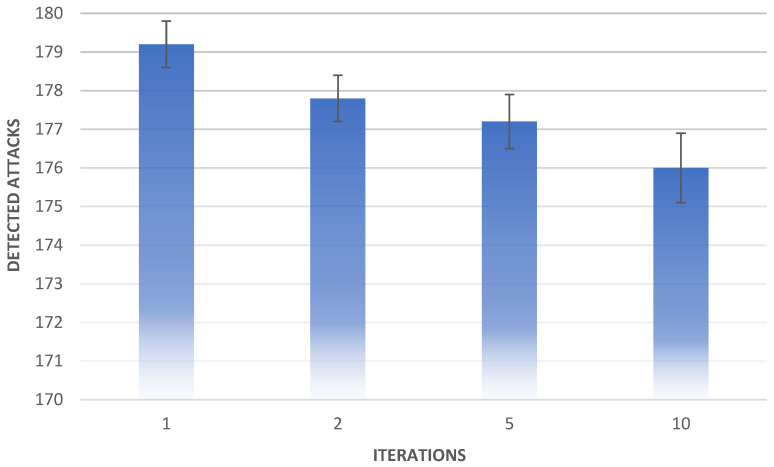
Effectiveness of intrusion detection for multi-iteration scenarios.

The authors performed numerous tests to show differences between different configurations of *Iterate_Snort* arguments; each test had a different number of iterations, but the total operation time was the same. We assumed that in a single full test, exactly 180 attacks should be executed (the script operated for approximately 15 min while performing 180 attacks). Therefore, we chose the duration of packet collection in each scenario because every restart of Snort takes some time; we took into account pauses between iterations and the time when commands are executed. The results are shown in Table 8 and Figure 4.

The comparative analysis shows that the detection algorithm is repeatable in terms of effectiveness when it comes to the same configuration. It is also worth mentioning that there is no significant drop in effectiveness between different scenarios. The difference between sample and ten-iteration tests’ effectiveness is lower than 2 percentage points. This means the shared data can be regularly updated and the algorithm would remain effective.

While the average number of attacks detected in a sample test (one iteration) is the highest, Snort does not always detect all of the attacks (the minimum number of attacks detected is always lower than 180). The two-iteration tests missed two attacks on average. The standard deviation of the results is less than 1 and there were no significant irregularities in the results (the difference between the maximum and minimum values of attacks detected is equal to 2). This suggests that increasing the number of iterations will lower effectiveness. The average number of attacks detected in the five-iteration test is indeed lower, although the standard deviation values mean the difference is inconclusive. The maximum number of attacks detected by the five-iteration test is 179 attacks. This means that under specific conditions (such as a short delay between starting the scripts or Snort initialisation time lower than usual), the algorithm can perform very well, even with a higher number of iterations. The average number of attacks detected by ten-iteration tests is lower than the result of the two-iteration tests. While the number of lost packets is higher than in previous tests, the algorithm still performs very well: its effectiveness is nearly 98% despite running ten iterations.

Two main conclusions can be drawn from this analysis.

- In most cases, the algorithm cases perform better with a lower number of iterations. Its effectiveness is higher in one-iteration scenarios than in two-, five-, and ten-iteration scenarios. The two-iteration test’s effectiveness is also higher than that of the ten-iteration tests.
- The standard deviation of the tests is lower than 1 in each scenario. This means that the algorithm regularly detects attacks, and anomalies such as the minimum number of attacks detected by the ten-iteration test (174 attacks) are rare throughout its operation.

## 6. Conclusions

Security in cyberspace is a major challenge of modern IT systems [63,64], driving the development of new ways of detecting and protecting against threats and attacks. This paper proposes a multivariable heuristic algorithm as a new method of intrusion detection. This solution is based on different types of flags and values of entropy set for each suspicious address. Such information about suspicious addresses can be shared between entities in a federated environment. This makes the algorithm flexible and adaptable to different sectors and networks, as the flags are changed within a single CSV file. Depending on the input data, the algorithm calculates the packet value and decides—depending on the sensitivity of the network (set by a variable defined in the shared file)—whether the packet should be reported. Additionally, the authors propose an approach to parametrise the detection algorithm. The authors propose default values of the packet_value, sensitivity, and entropy variables in case these values are not set by the user manually, since they are crucial for the operation of the algorithm.

The effectiveness of the proposed solution was verified through a series of tests with different configurations. Snort—a popular open source IDS tool—was used during the experiments. The authors implement new functionalities in this environment to verify the introduced multivariable heuristic detection algorithm. The testing part consisted of two scenarios: functionality verification and comparative analysis of the algorithm. The total time was the same in each case, but the number of algorithm iterations increased. In each test, the values of the flags and entropy were random to show that the algorithm is effective in different network scenarios. The first scenario validated the algorithm operation: the decision based on the value of the packet counted using the proposed formula was made correctly. The second scenario was performed to check how the changes of detection duration affect the effectiveness. The authors drew two main conclusions: the algorithm performs better with a lower number of iterations, and it is rather repetitive in the same configuration.

With Internet use increasing rapidly every day, solutions such as the multivariable heuristic intrusion detection algorithm are highly desirable on the market. The new algorithm proposed in this paper was tested and its effectiveness demonstrated, although it is still open for future development. Some environments may need additional sector-specific groups of flags that describe the character of a given threat. Another potential extension that could increase network security is collecting additional information about traffic using network devices, e.g., by monitoring the number of inbound packets on firewalls. These statistics could help prevent DoS and DDoS attacks more effectively. Future work will explore these directions of development of the multivariable-based approach to intrusion detection. The research will also focus on finding the optimal default parameters of the heuristic algorithm for different sectors. Such personalised parameters will increase the effectiveness of threat detection in a given network. It is important that the development of detection methods continues, given the fact that new threats and attacks are constantly appearing in cyberspace.

The following abbreviations are used in this manuscript:

## Figures and Tables

**Table 1:** Description of dangerous flags.

Flag Type	Flag	Description	Default Value
dangerous	H (High)	High threat	−6
dangerous	M (Medium)	Medium threat	−5
dangerous	L (Low)	Low threat	−2

**Table 2:** Description of attack flags.

Flag Type	Flag	Description	Default Value
attack	D	DDoS	−5
attack	P	Phishing	−5
attack	M	Malware	−5
attack	R	Ransomware	−5
attack	S	DoS	−5
attack	X	XSS	−5

**Table 3:** Description of range flags.

Flag Type	Flag	Description	Default Value
range	S (Single)	Attack targeting the attacked station only	−1
range	P (Partial)	Attack on stations affecting adjacent components	−2
range	C (Complete)	Attack on stations affecting the entire network	−3

**Table 4:** Description of access flags.

Flag Type	Flag	Description	Default Value
access	N (None)	Attack does not require user response inside the network	−2
access	U (User)	Attack requires user response inside the network	−1

**Table 5:** Description of availability flags.

Flag Type	Flag	Description	Default Value
availability	N (None)	Attack does not affect the functionality of the station	−1
availability	P (Partial)	Attack causes the loss of several functionalities and a decrease in performance	−2
availability	C (Complete)	Attack causes a complete loss of control of the unit	−4

**Table 6:** Default values of parameters.

Parameter	Default Value
α	0.65
β	0.35
γ	1
δ	1
ϵ	1
η	0.5

**Table 7:** Default values regarding detection threshold.

Parameter	Default Value
packet_value	23
sensitivity	15
entropy	5

**Table 8:** Effectiveness for multi-iteration scenarios.

Number of Iterations	Maximum Attacks Detected	Minimum Attacks Detected	Average Attacks Detected	Effectiveness	Standard Deviation
1	180	178	179.2	99.6%	0.6
2	179	177	177.8	98.8%	0.6
5	179	176	177.2	98.4%	0.7
10	177	174	176.0	97.8%	0.9

## References

[1] Sajal S.Z., Jahan I., Nygard K.E. A Survey on Cyber Security Threats and Challenges in Modem Society. Proceedings of the 2019 IEEE International Conference on Electro Information Technology (EIT).

[2] Hussain A., Mohamed A., Razali S. (2020). A Review on Cybersecurity: Challenges & Emerging Threats. Proceedings of the 3rd International Conference on Networking, Information Systems & Security, NISS2020.

[3] Kettani H., Wainwright P. On the Top Threats to Cyber Systems. Proceedings of the 2019 IEEE 2nd International Conference on Information and Computer Technologies (ICICT).

[4] Aiyanyo I.D., Samuel H., Lim H. (2020). A Systematic Review of Defensive and Offensive Cybersecurity with Machine Learning. Appl. Sci..

[5] Cyber Security Statistics (2021). The Ultimate List Of Stats, Data & Trends. https://purplesec.us/resources/cyber-security-statistics/.

[6] Ransomware Statistics (2020). Trends and Facts for 2020 and Beyond. https://www.cloudwards.net/ransomware-statistics/.

[7] Whitman M.E., Mattord H.J. (2011). Principles of Information Security.

[8] Ramapantulu L., Teo Y.M., Chang E. A conceptural framework to federate testbeds for cybersecurity. Proceedings of the 2017 Winter Simulation Conference (WSC).

[9] Shaked A., Tabansky L., Reich Y. (2020). Incorporating systems thinking into a cyber resilience maturity model. IEEE Eng. Manag. Rev..

[10] Cybersecurity Competence Network. https://cybercompetencenetwork.eu.

[11] ECHO Project Portal. https://echonetwork.eu/project-summary/.

[12] Al-Asli M., Ghaleb T.A. Review of Signature-based Techniques in Antivirus Products. Proceedings of the 2019 International Conference on Computer and Information Sciences (ICCIS).

[13] Samrin R., Vasumathi D. Review on anomaly based network intrusion detection system. Proceedings of the 2017 International Conference on Electrical, Electronics, Communication, Computer, and Optimization Techniques (ICEECCOT).

[14] Paulauskas N., Baskys A. (2019). Application of Histogram-Based Outlier Scores to Detect Computer Network Anomalies. Electronics.

[15] Kenny V., Nathal M., Saldana S. (2014). Northwestern University Open Text Book on Process Optimization— Heuristic Algorithms. https://optimization.mccormick.northwestern.edu/index.php/Heuristic_algorithms.

[16] Ali W., Malebary S. (2020). Particle Swarm Optimization-Based Feature Weighting for Improving Intelligent Phishing Website Detection. IEEE Access.

[17] Jacob B. (2011). Automatic XSS Detection and Snort Signatures/ACLs Generation by the Means of a Cloud-Based Honeypot System. Master’s Thesis.

[18] Yerong T., Sai S., Ke X., Zhe L. Intrusion Detection Based on Support Vector Machine Using Heuristic Genetic Algorithm. Proceedings of the 2014 Fourth International Conference on Communication Systems and Network Technologies.

[19] Jothi K.R., Balaji B S., Pandey N., Beriwal P., Amarajan A. An Efficient SQL Injection Detection System Using Deep Learning. Proceedings of the 2021 International Conference on Computational Intelligence and Knowledge Economy (ICCIKE).

[20] Rajesh M. (2021). Intensive analysis of intrusion detection methodology over Mobile Adhoc Network using machine learning strategies. Mater. Today Proc..

[21] Bangui H., Buhnova B. (2021). Recent Advances in Machine-Learning Driven Intrusion Detection in Transportation: Survey. Procedia Comput. Sci..

[22] Saravanan L., Himanshu S., Sreenivasulu K., Deivakani M. (2021). Detection of software intrusion based on machine learning techniques for IOT systems. Mater. Today Proc..

[23] Kalimuthan C., Arokia Renjit J. (2020). Review on intrusion detection using feature selection with machine learning techniques. Mater. Today Proc..

[24] Kilincer I.F., Ertam F., Sengur A. (2021). Machine learning methods for cyber security intrusion detection: Datasets and comparative study. Comput. Netw..

[25] Fang W., Tan X., Wilbur D. (2020). Application of intrusion detection technology in network safety based on machine learning. Saf. Sci..

[26] Mahboob A.S., Moghaddam M.R.O. An Anomaly-based Intrusion Detection System Using Butterfly Optimization Algorithm. Proceedings of the 2020 6th Iranian Conference on Signal Processing and Intelligent Systems (ICSPIS).

[27] Luo H., Shi K., Qiao F., Li Y. Intrusion Detection Mechanism Based On Modular Neural Network. Proceedings of the 2020 2nd International Conference on Machine Learning, Big Data and Business Intelligence (MLBDBI).

[28] Lin Z., Hongle D. Research on SDN intrusion detection based on online ensemble learning algorithm. Proceedings of the 2020 International Conference on Networking and Network Applications (NaNA).

[29] Jain V., Agrawal M. Applying Genetic Algorithm in Intrusion Detection System of IoT Applications. Proceedings of the 2020 4th International Conference on Trends in Electronics and Informatics (ICOEI)(48184).

[30] Saravanan K., Subburathinam K. (2012). Packet Score based network security and Traffic Optimization. arXiv.

[31] Murtuza S., Asawa K. Mitigation and Detection of DDoS Attacks in Software Defined Networks. Proceedings of the 2018 Eleventh International Conference on Contemporary Computing (IC3).

[32] Prasath M.K., Perumal B. (2019). A meta-heuristic Bayesian network classification for intrusion detection. Int. J. Netw. Manag..

[33] Umbarkar S., Shukla S. Analysis of Heuristic based Feature Reduction method in Intrusion Detection System. Proceedings of the 2018 5th International Conference on Signal Processing and Integrated Networks (SPIN).

[34] Manzoor I., Kumar N. (2017). A feature reduced intrusion detection system using ANN classifier. Expert Syst. Appl..

[35] Mukhopadhyay I., Gupta K.S., Sen D., Gupta P. Heuristic Intrusion Detection and Prevention System. Proceedings of the 2015 International Conference and Workshop on Computing and Communication (IEMCON).

[36] Varma P.R.K., Kumari V.V., Kumar S.S. (2016). Feature Selection Using Relative Fuzzy Entropy and Ant Colony Optimization Applied to Real-time Intrusion Detection System. Procedia Comput. Sci..

[37] Xing H.J., Ren H.R. (2014). Regularized correntropy criterion based feature extraction for novelty detection. Neurocomputing.

[38] Pivarníková M., Sokol P., Bajtoš T. (2020). Early-Stage Detection of Cyber Attacks. Information.

[39] Scarfone K., Mell P. (2012). Guide to Intrusion Detection and Prevention Systems (IDPS).

[40] Stallings W. (2011). Cryptography and Network Security: Principles and Practice.

[41] Arshad J., Azad M.A., Amad R., Salah K., Alazab M., Iqbal R. (2020). A Review of Performance, Energy and Privacy of Intrusion Detection Systems for IoT. Electronics.

[42] Beale J. (2004). Snort 2.1 Intrusion Detection.

[43] Papadogiannaki E., Ioannidis S. (2021). Acceleration of Intrusion Detection in Encrypted Network Traffic Using Heterogeneous Hardware. Sensors.

[44] Soniya S.S., Vigila S.M.C. Intrusion detection system: Classification and techniques. Proceedings of the 2016 International Conference on Circuit, Power and Computing Technologies (ICCPCT).

[45] Aryachandra A.A., Arif Y.F., Anggis S.N. Intrusion Detection System (IDS) server placement analysis in cloud computing. Proceedings of the 2016 4th International Conference on Information and Communication Technology (ICoICT).

[46] Snort_Team SNORT^®^ Users Manual 2.9.16. http://manual-snort-org.s3-website-us-east-1.amazonaws.com.

[47] Suricata—Open Source IDS/IPS/NSM Engine. https://suricata-ids.org.

[48] Cisco Systems Security Products. www.cisco.com/c/en/us/products/security/firewalls.

[49] Palo Alto Networks Security Products. www.paloaltonetworks.com/network-security.

[50] Oliveira N., Praça I., Maia E., Sousa O. (2021). Intelligent Cyber Attack Detection and Classification for Network-Based Intrusion Detection Systems. Appl. Sci..

[51] Kim G., Lee S., Kim S. (2014). A novel hybrid intrusion detection method integrating anomaly detection with misuse detection. Expert Syst. Appl..

[52] Li J., Li Q., Zhou S., Yao Y., Ou J. A review on signature-based detection for network threats. Proceedings of the 2017 IEEE 9th International Conference on Communication Software and Networks (ICCSN).

[53] Kim J., Park M., Kim H., Cho S., Kang P. (2019). Insider Threat Detection Based on User Behavior Modeling and Anomaly Detection Algorithms. Appl. Sci..

[54] Lyda R., Hamrock J. (2007). Using Entropy Analysis to Find Encrypted and Packed Malware. IEEE Secur. Priv..

[55] Menéndez H.D., Clark D.T., Barr E. (2021). Getting Ahead of the Arms Race: Hothousing the Coevolution of VirusTotal with a Packer. Entropy.

[56] Hemalatha J., Roseline S.A., Geetha S., Kadry S., Damaševičius R. (2021). An Efficient DenseNet-Based Deep Learning Model for Malware Detection. Entropy.

[57] Khamphakdee N., Benjamas N., Saiyod S. (2015). Improving Intrusion Detection System Based on Snort Rules for Network Probe Attacks Detection with Association Rules Technique of Data Mining. J. ICT Res. Appl..

[58] Coşar M., Kiran H.E. Performance Comparison of Open Source IDSs via Raspberry Pi. Proceedings of the 2018 International Conference on Artificial Intelligence and Data Processing (IDAP).

[59] Caswell B., Beale J., Baker A. (2007). Snort Intrusion Detection and Prevention Toolkit.

[60] Jin S., Li M., Wang Z. Research and Design of Preprocessor Plugin Based on PCRE under Snort Platform. Proceedings of the 2011 International Conference on Control, Automation and Systems Engineering (CASE).

[61] Audi-1 GitHub, SQLI Labs. github.com/Audi-1/sqli-labs.

[62] Rinkish GitHub, Sqli_Edited_Version. github.com/Rinkish/Sqli_Edited_Version.

[63] Ani U.P.D., He H.M., Tiwari A. (2017). Review of cybersecurity issues in industrial critical infrastructure: Manufacturing in perspective. J. Cyber Secur. Technol..

[64] Mohamed N., Al-Jaroodi J., Jawhar I. Opportunities and Challenges of Data-Driven Cybersecurity for Smart Cities. Proceedings of the 2020 IEEE Systems Security Symposium (SSS).

